# Inhibition of the Aquaporin-1 Cation Conductance by Selected Furan Compounds Reduces Red Blood Cell Sickling

**DOI:** 10.3389/fphar.2021.794791

**Published:** 2022-01-17

**Authors:** Pak Hin Chow, Charles D. Cox, Jinxin V. Pei, Nancy Anabaraonye, Saeed Nourmohammadi, Sam W. Henderson, Boris Martinac, Osheiza Abdulmalik, Andrea J. Yool

**Affiliations:** ^1^ Aquaporin Physiology and Drug Discovery Program, School of Biomedicine, University of Adelaide, Adelaide, SA, Australia; ^2^ Victor Chang Cardiac Research Institute, Darlinghurst, NSW, Australia; ^3^ St Vincent’s Clinical School, University of New South Wales, Darlinghurst, NSW, Australia; ^4^ Research School of Biology, College of Science, Australian National University, Canberra, ACT, Australia; ^5^ Division of Hematology, Children’s Hospital of Philadelphia, Philadelphia, PA, United States

**Keywords:** AQP1, cyclic GMP, Piezo1 channel, psickle, erythrocyte, RBC, sickle cell anemia

## Abstract

In sickle cell disease (SCD), the pathological shift of red blood cells (RBCs) into distorted morphologies under hypoxic conditions follows activation of a cationic leak current (Psickle) and cell dehydration. Prior work showed sickling was reduced by 5-hydroxylmethyl-2-furfural (5-HMF), which stabilized mutant hemoglobin and also blocked the Psickle current in RBCs, though the molecular basis of this 5-HMF-sensitive cation current remained a mystery. Work here is the first to test the hypothesis that Aquaporin-1 (AQP1) cation channels contribute to the monovalent component of Psickle. Human AQP1 channels expressed in *Xenopus* oocytes were evaluated for sensitivity to 5-HMF and four derivatives known to have differential efficacies in preventing RBC sickling. Ion conductances were measured by two-electrode voltage clamp, and osmotic water permeability by optical swelling assays. Compounds tested were: 5-HMF; 5-PMFC (5-(phenoxymethyl)furan-2-carbaldehyde); 5-CMFC (5-(4-chlorophenoxymethyl)furan-2-carbaldehyde); 5-NMFC (5-(2-nitrophenoxymethyl)-furan-2-carbaldehyde); and VZHE006 (tert-butyl (5-formylfuran-2-yl)methyl carbonate). The most effective anti-sickling agent, 5-PMFC, was the most potent inhibitor of the AQP1 ion conductance (98% block at 100 µM). The order of sensitivity of the AQP1 conductance to inhibition was 5-PMFC > VZHE006 > 5-CMFC ≥ 5-NMFC, which corresponded with effectiveness in protecting RBCs from sickling. None of the compounds altered AQP1 water channel activity. Combined application of a selective AQP1 ion channel blocker AqB011 (80 µM) with a selective hemoglobin modifying agent 5-NMFC (2.5 mM) increased anti-sickling effectiveness in red blood cells from human SCD patients. Another non-selective cation channel known to be expressed in RBCs, Piezo1, was unaffected by 2 mM 5-HMF. Results suggest that inhibition of AQP1 ion channels and capacity to modify hemoglobin are combined features of the most effective anti-sickling agents. Future therapeutics aimed at both targets could hold promise for improved treatments for SCD.

## 1 Introduction

Sickle cell disease (SCD) results from an inherited mutation in the oxygen-carrying molecule hemoglobin in red blood cells. Unlike wild type hemoglobin (Hb), hemoglobin carrying the SCD mutation (HbS) polymerizes more readily in low oxygen conditions into stiff strands which distort red blood cell morphology into diagnostic dysfunctional shapes. The SCD single point mutation in HbS converts a key glutamate residue to valine (Pauling et al., 1949; Eaton 2020). When deoxygenated, HbS molecules aggregate into rigid polymers, changing red blood cell shape, and increasing fragility, solute loss and stickiness (Pauling et al., 1949; Joiner 1993). Clinical concerns include chronic anemia, acute ischemia, severe pain episodes, and organ damage (Steinberg 1999; Rees et al., 2010). Current interventions include blood transfusions to alleviate symptoms, and hydroxyurea treatment to reduce pain crises and anemia, but chronic side effects and variability in individual responsiveness to hydroxyurea limit its usefulness (Perutz and Mitchison 1950; Charache et al., 1987; Segal et al., 2008). Transplanting stem cells from donor bone marrow is the only cure for SCD (Saraf and Rondelli 2019), an option not available to most patients around the world.

Pharmacological treatment strategies have focused on identifying chemical modifiers to stabilize HbS. Promising compounds such as aromatic aldehydes and benzaldehydes have been tested in sickle cells (Zaugg et al., 1977; Beddell et al., 1984; Abraham et al., 1991). 5-Hydroxymethyl-2-furfural (5-HMF) forms a Schiff base with HbS, increasing oxygen affinity, reducing polymerization risk, and protecting red blood cells from sickling (Abdulmalik et al., 2005). Building on this discovery, Abdulmalik, Safo and others (Xu et al., 2017) engineered a group of ester and ether derivatives of 5-HMF which they tested for effectiveness in modifying Hb, improving oxygen affinity, and preventing hypoxia-induced sickling of human SCD red blood cells. Of interest, four of the 12 synthesized compounds conferred better protection from sickling than 5-HMF (at 2 mM) and were effective in modifying hemoglobin, pointing to a new generation of candidate therapeutics. Curiously, some derivatives in the group such as 5-NFMC and 5-CMFC (despite yielding significant modification of Hb), were similar or worse than 5-HMF in preventing sickling (Zaugg et al., 1977; Xu et al., 2017). We considered this a clue that effective anti-sickling agents might serendipitously be affecting secondary targets, in addition to hemoglobin.

Volume regulation in RBCs involves a network of ion and water transport mechanisms which enable careful control of hemoglobin concentration within a narrow window (Gallagher 2017). In the resting state, normal RBC membrane cation permeability is low, but increases in cation fluxes are evident during SCD cell sickling, mediated by the K^+^-Cl^−^ cotransporter (KCC), the Ca^2+^-activated K^+^ (Gardos) channel, and a less well understood leak pathway known as “Psickle” (Joiner 1993; Lew and Bookchin 2005). The involvement of a cation leak current in the sickling process has been appreciated for decades based on imbalances in K^+^ and Na^+^ levels in SCD cells (Tosteson et al., 1952). However, a mystery concerning the molecular basis of Psickle has persisted, despite widespread interest in finding a Psickle inhibitor for expanding therapeutic strategies (Lew et al., 1997; Hannemann et al., 2014; Al Balushi et al., 2019; Kaestner et al., 2020). Cation leak currents are activated in sickle cell RBCs during deoxygenation (Joiner 1993; Joiner et al., 1988; Ma et al., 2012) and can be potentiated by membrane shear stress (Johnson and Gannon 1990), such as that experienced in the microcirculation (McMahon 2019).

AQP1 is expressed in red blood cells (Ma et al., 1998; Maeda et al., 2009), and tissues including kidney, vascular system, heart, brain and others (Venero et al., 2001; Badaut et al., 2002; Papadopoulos et al., 2002; Speake et al., 2003; Montiel et al., 2020). AQP1 serves dual roles as a water channel and a non-selective cation channel regulated by cGMP acting at the loop D domain (Yu et al., 2006; Kourghi et al., 2018). Although initially controversial, converging lines of evidence have shown AQP1 conducts non-selective monovalent cations via the central pore of the tetramer, which is functionally and pharmacologically distinct from the water-selective pores in the monomeric subunits (Anthony et al., 2000; Saparov et al., 2001; Boassa and Yool, 2003; Yu et al., 2006; Zhang et al., 2007). As reviewed elsewhere, other classes of AQPs also function as ion channels including AQP0, AQP6, *Drosophila* Big Brain, soybean Nodulin and *Arabidopsis* PIP2; 1 (Yool and Campbell 2012; Tyerman et al., 2021).

A possible role for AQP1 ion channels as the monovalent component of the Psickle conductance has not previously been considered. Recent work demonstrated that 5-HMF caused dose-dependent block of the AQP1 ion conductance, inducing 90% block of the cGMP activated ion current at 10 mM, 74% at 1 mM and 43% at 0.5 mM, with no effect on AQP1-mediated water fluxes (Chow et al., 2020), supporting the hypothesis tested here that the furan derivatives which are effective in reducing red blood cell sickling also act as pharmacological inhibitors of the AQP1 cation conductance.

In summary, results here showed two furan derivatives that are effective in preventing SCD cell sickling, 5-PMFC and VZHE006, significantly inhibited the AQP1 cation conductance, as did the parent compound 5-HMF. No inhibition of the AQP1 ion conductance was observed with 5-NMFC or 5-CMFC, which modified hemoglobin but were less effective in reducing sickling. Piezo1, a mechanosensitive cation channel proposed to mediate Psickle in RBCs, is unlikely to account in full for the Psickle current, since it was not affected by 5-HMF. However, a further role for Piezo1 in Ca^2+^ entry associated with the Psickle current and RBC dehydration seems likely. AQP1 ion channels are selective for monovalent cations, not Ca^2+^ (Yool et al., 1996), whereas Piezo1 enables Ca^2+^ permeation (Gnanasambandam et al., 2015). To test the idea that effective anti-sickling furan agents acted on both HbS and AQP1, red blood cells from human SCD patients were tested with 5-NMFC which modifies Hb, in combination with the bumetanide derivative AqB011 which blocks AQP1 ion channels (Kourghi et al., 2016). Results showed that combined agents increased the duration of protection from sickling in human SCD cells in hypoxic conditions as compared to either agent alone, and approached the protective effect of 5-PMFC which affects both targets simultaneously.

This work is the first to identify AQP1 as a molecular component of the monovalent Psickle current, and to establish AQP1 as a therapeutic target for consideration in the development of anti-sickling treatments. In practice, useful therapeutic agents would not need to prevent sickling completely, but simply would need to slow the process of HbS polymerization sufficiently that a larger proportion of RBCs could successfully pass through the microcirculation (Eaton 2020). Novel therapeutic agents with combined actions on AQP1 ion channel inhibition and HbS stabilization could offer the desired slowing of dehydration and HbS polymerization, a goal of international interest for expanding the range of affordable clinical options for treating sickle cell disease.

## 2 Materials and Methods

### 2.1 Furan Compounds

5-HMF (5-hydroxymethyl-2-furfural); 5-PMFC (5-(phenoxymethyl)furan-2-carbaldehyde); 5-CMFC (5-(4-chlorophenoxymethyl)furan-2-carbaldehyde); and 5-NMFC (5-(2-nitrophenoxy methyl)-furan-2-carbaldehyde) were purchased from Sigma Aldrich Chemicals (MO, United States). VZHE006 (tert-butyl (5-formylfuran-2-yl)methyl carbonate), synthesized at Virginia Commonwealth University (United States) as described previously (Xu et al., 2017) was kindly provided by Dr Martin Safo. 5-HMF was dissolved in water, and other compounds were dissolved in dimethylsulfoxide (DMSO) to create 1,000x stock solutions, which were diluted 1 µl/ml into experimental salines to final concentrations. The AQP1 ion channel antagonist AqB011 (Kourghi et al., 2016) also was made as a 1,000x stock solution in DMSO for dilution in experimental salines. Pharmacological agents as powders were stored dry at −20°C, resuspended as stock solutions in DMSO and kept at 4°C for up to 8 months, and diluted in fresh saline prior to experimental use. Equivalent amounts of DMSO alone in saline served as the vehicle control.

### 2.2 Oocyte Preparation and Injection

Unfertilized oocytes were harvested from *Xenopus laevis* frogs in accord with national guidelines (Australian Code of Practice for the Care and Use of Animals for Scientific Purposes), using a protocol approved by the University of Adelaide Animal Ethics Committee (#M2018-016). Oocytes were defolliculated with collagenase type 1A (2 mg/ml) in isotonic saline (100 mM NaCl, 2 mM KCl, 5 mM MgCl_2_, and 5 mM HEPES; pH 7.6) at approximately 16–18°C for 1.5 h. Oocytes were washed 3–4 times at ∼10 min intervals with isotonic saline and kept at 18°C in frog Ringers saline (isotonic saline supplemented with 0.6 mM CaCl_2_, 5% horse serum (v/v), 100 units/ml penicillin, 0.1 mg/ml streptomycin, and 0.5 mg/ml tetracycline). Human AQP1 (Genbank # NM_--_198098) and human AQP4 (Genbank #NM_--_001650, OriGene) genes were subcloned into a *Xenopus ß*-globin expression vector (Anthony et al., 2000). AQP1 cDNA was linearized with BamHI and cRNA was synthesized *in vitro* with the T3 mMessage mMachine kit (Invitrogen, United States). Human AQP4 cDNA was linearized with NheI-HF (BioLabs) and synthesized *in vitro* with the T7 mMessage mMachine Kit. RNAs were resuspended in sterile water and stored at −80°C. Oocytes were injected with 50 nl of sterile water (sham injection) or 50 nl of water containing 1 ng of AQP1 or AQP4 wild type cRNA. Injected oocytes were incubated in frog Ringer’s saline at 18°C for 48 h or more to allow time for protein expression. All chemicals were from Sigma-Aldrich (St. Louis, MO United States), unless otherwise indicated.

### 2.3 Quantitative Swelling Assays

Prior to swelling assays, sham-injected (non-AQP1 controls) and AQP1-expressing oocytes were rinsed in isotonic saline (without serum, antibiotic-free) for at least 1 h at room temperature. Swelling rates were measured in 50% hypotonic saline (isotonic Na^+^ saline diluted with an equal volume of water, without test compounds present) and quantified by changes in the oocyte cross-sectional area imaged by videomicroscopy (Cohu, CA United States) at 1 frame per second for 30–60 s, using NIH ImageJ software (National Institutes of Health, MD United States). In double-swelling assays, swelling was first measured without drug treatment (first swelling “S1”). Oocytes were then incubated for 2 h in isotonic saline with vehicle or one of the test compounds, and reassessed in a second swelling assay (“S2”) in hypotonic saline without pharmacological agents, as described previously (Migliati et al., 2009). Swelling rates were measured from slopes of linear regression fits of relative volume as a function of time using Prism software (GraphPad Inc., CA United States).

### 2.4 Electrophysiology

Two-electrode voltage clamp recordings of non-AQP1 control and AQP1-expressing oocytes in isotonic Na^+^ saline were done with capillary glass electrodes (1–3 MΩ) filled with 1 M KCl, using a GeneClamp 500B (Molecular Devices, CA United States) amplifier. Bath application of membrane permeable 8CPT-cGMP (8-(4-chlorophenylthio)-guanosine 3′,5′-cyclic monophosphate) at 10 µM final or SNP (sodium nitroprusside) at 1–3 mM final was used to activate the ionic conductance in AQP1-expressing oocytes, as described previously (Campbell et al., 2012; Kourghi et al., 2018). Changes in current as a function of time were monitored by repeated +40 mV voltage steps from a holding potential of −40 every 6 s. Conductance values were measured as the slope of linear fits (from −40 to +60 mV) using a current-voltage protocol with steps from +60 to −110 or −120 mV. Recordings were filtered at 2 kHz, and stored to hard disk for offline analysis. Data were analyzed with Clampex 9.0 software (pClamp 9.0, Molecular Devices, CA United States) and Prism software.

Piezo1^−/−^ HEK293T cells (a kind gift from Ardem Patapoutian) stably transfected with human Piezo1 were plated on 35 mm dishes for patch-clamp analysis. The extracellular solution for cell-attached patches consisted of 90 mM potassium aspartate, 50 mM KCl, 1 mM MgCl_2_ and 10 mM HEPES (pH 7.2; adjusted using KOH). The pipette solution contained 140 mM NaCl, 3 mM KCl, 1 mM CaCl_2_, 1 mM MgCl_2_ with 10 mM HEPES (pH 7.2; adjusted using NaOH). Negative pressure was applied to patch pipettes using a High-Speed Pressure Clamp-1 (ALA Scientific Instruments) and recorded in millimeters of mercury (mmHg) using a piezoelectric pressure transducer (WPI, Sarasota, FL, United States). Borosilicate glass pipettes (Sigma, St Louis, MO, United States) were pulled using a vertical pipette puller (PP-83, Narashige, Japan) to produce electrodes with resistances of 2.0–2.3 MΩ. Single-channel Piezo1 currents were amplified using an AxoPatch 200B amplifier (Axon Instruments), and data were acquired at a sampling rate of 10 kHz with 1 kHz filtration and analyzed using pCLAMP10 software (Axon Instruments). Boltzmann distribution functions were used to analyze dependence of mesoscopic Piezo1 channel currents and open probability on the negative pressure applied via patch pipettes. The Boltzmann plots were obtained by fitting open probability *P*_o_∼*I*/*I*_max_ versus negative pressure using the expression *P*_o_/(1–*P*_o_) = exp [*α*(***P***–*P*_1/2_)], where ***P*** is the negative pressure (suction) [mmHg], *P*_1/2_ is the negative pressure at which *P*_o_ = 0.5, and *α* [mmHg^−1^] is the slope of the plot ln [*P*_o_/(1–*P*_o_) = [*α*(***P***–*P*_1/2_)] reflecting the channel mechanosensitivity.

### 2.5 Analyses of Sickling in Human SDC Red Blood Cells

Blood samples from homozygous SCD patients were used with informed consent in accord with an Institutional Review Board approved protocol for research involving human subjects at the Children’s Hospital of Philadelphia. Blood suspensions (hematocrit values of 20%) were incubated under air with and without 80 µM AqB011 and furan compounds 5-NMFC and 5-PMFC at 2.5 mM concentrations at 37°C for 1 h, then subjected to hypoxia (100% nitrogen) at 37°C for 1 h. Samples were fixed without exposure to air in buffered 2% glutaraldehyde solution, and imaged by microscope for quantitative assessment of the percentages of cells showing sickling morphologies in the treatment conditions. The percentages of sickled cells were determined using validated NIH ImageJ machine-learning software analyses of microscopic images, a method enabling reliable and reproducible detection of sickled shapes based on a combination of circular and elliptical shape factors, as previously reported (Abdulmalik et al., 2005; Xu et al., 2017).

### 2.6 *In Silico* Docking Modeling

*In silico* modeling was carried out using methods successfully employed previously to identify candidate sites for pharmacological modulators of AQP1 channels (Kourghi et al., 2016). The human AQP1 protein crystal structure (PDB ID:1FQY) was obtained from the NIH National Center for Biotechnology Information Structure database (available at www.ncbi.nlm.nih.gov/Structure/pdb/1FQY). Structures for 5-HMF and the derivatives were downloaded from NIH PubChem (pubchem.ncbi.nlm.nih.gov) and converted into software-compatible 3D structures in. pdb format using the online SMILES Translator and Structure File Generator (National Cancer Institute, U.S. Department Health and Human Services, Washington DC). MGLtools was used for preparing both AQP1 and ligand docking coordinates. The docking was performed using Autodock Vina (Trott and Olson 2010), setting the docking grid to cover the intracellular face of the tetrameric pore.

### 2.7 Data Analysis and Statistics

Results compiled from replicate experiments are presented as box plots to show the full range of data points. Boxes represent 50% of the data points; the error bars indicate the full ranges; horizontal bars are median values. Statistically significant differences were evaluated as indicated in the Figure legends.

## 3 Results

**FIGURE 1:**
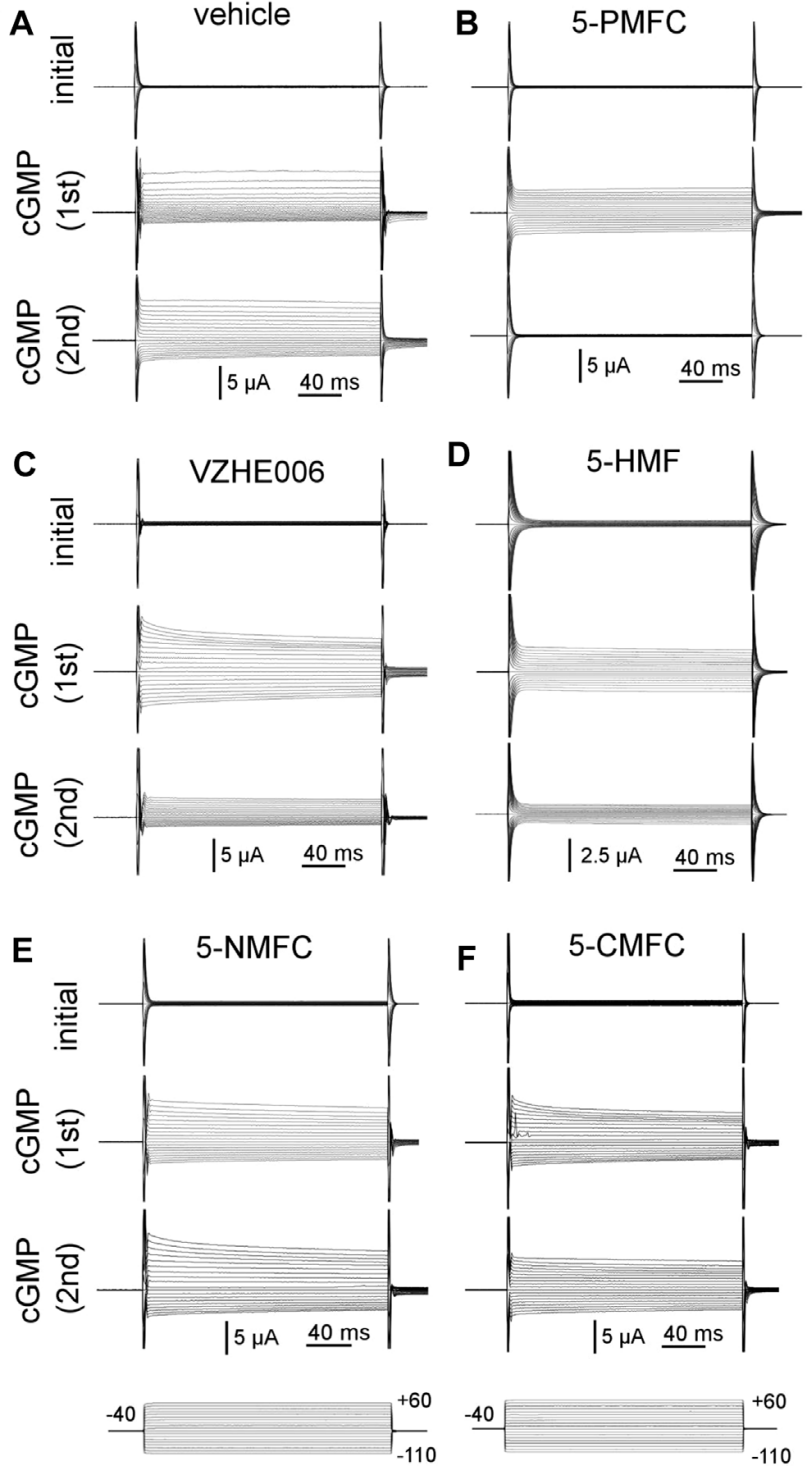
Electrophysiological recordings illustrating the effects of furan derivatives on the 8CPT-cGMP-activated AQP1 ion conductance. Representative sets of traces recorded by two-electrode voltage clamp of AQP1-expressing oocytes showing the initial conductance; the response induced by the first application of membrane-permeable 8CPT-cGMP; and the response to a second application of cGMP after 2 h of incubation in isotonic saline containing **(A)** equivalent DMSO vehicle; and 0.5 mM of **(B)** 5-PMFC; **(C)** VZHE006; **(D)** 5-HMF; **(E)** 5-NMFC; **(F)** 5-CMFC.

Electrophysiological analyses tested the effects of four furan derivatives, 5-PMFC, VZHE006, 5-NMFC, and 5-CMFC at 0.5 mM each, on the 8CTP-cGMP-activated ionic conductance in human AQP1-expressing oocytes (Figure 1), and confirmed block by the original scaffold compound 5-HMF at 0.5 mM. Currents were measured before (“initial”) and after activation by the cyclic GMP agonist (“cGMP 1st”), at approximately 30 min for 8CPT-cGMP (Campbell et al., 2012) or 10–15 min for SNP (Kourghi et al., 2018). After the initial recording, oocytes were removed from the bath chamber, then incubated for 2 h in isotonic saline with vehicle or the indicated agents, and then returned to the bath chamber for another set of voltage clamp recordings of responses to the second application of cGMP agonist (“cGMP 2nd”) without furans present in the bath saline. Prior work demonstrated that the establishment of AQP1 block by 5HMF was time-dependent, and required 1 h pre-incubation for full inhibition (Chow et al., 2020). Consistent with an intracellular site of action, the block was slowly reversible, showing recovery over hours after washout. Incubation with vehicle did not impair subsequent conductance responses of AQP1-expressing oocytes to cGMP agonists, which remained comparable in amplitude in repeated trials. Conductance responses were differentially inhibited following incubation in 0.5 mM 5-HMF, 5-PMFC, or VZHE006. No appreciable changes in responsiveness to cGMP were observed after incubation with 0.5 mM 5-NMFC or 5-CMFC.

**FIGURE 2:**
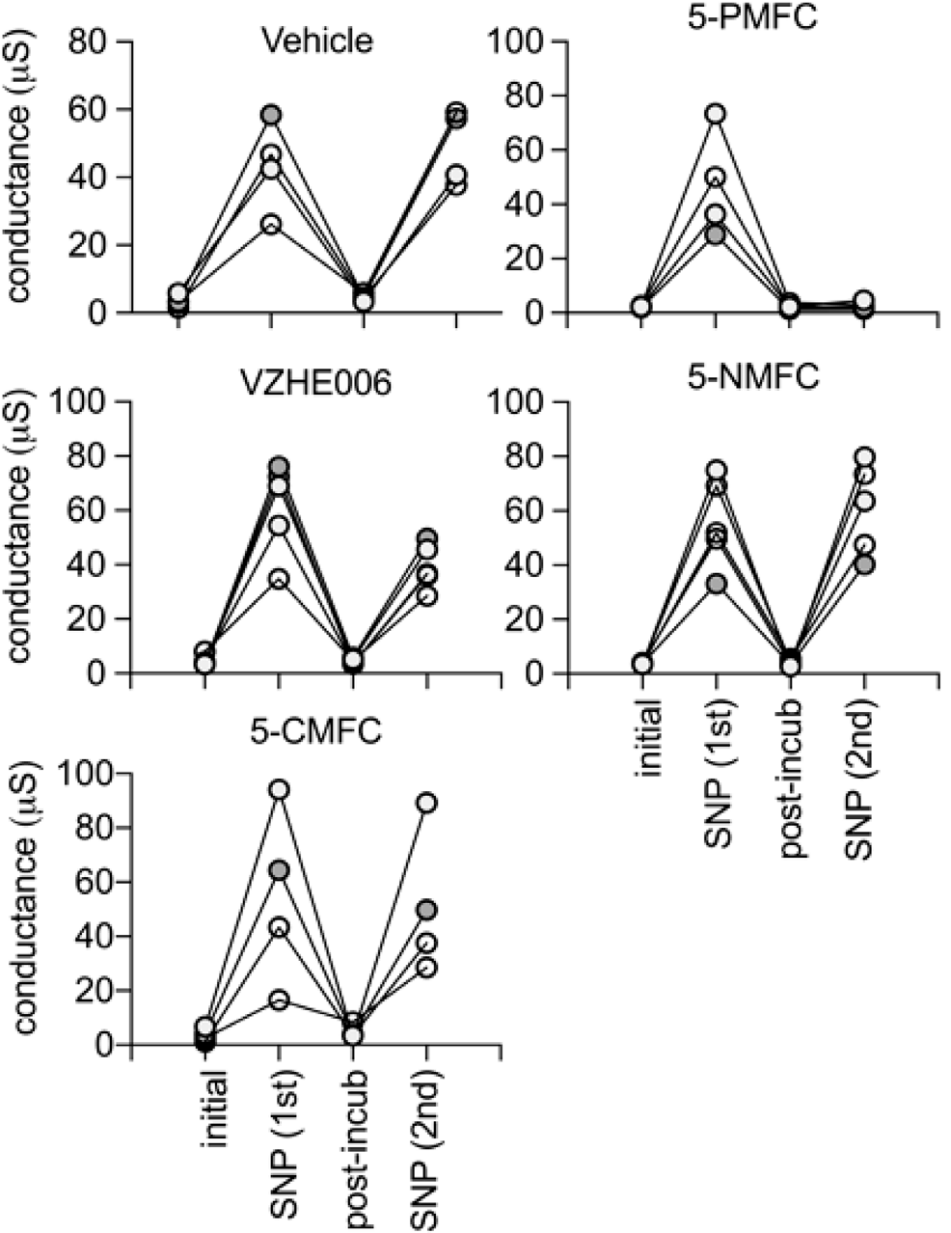
Trend plots showing the amplitude of the ionic currents, before (initial) and after the first activation by SNP (1st), the recovery after 2 h incubation with the indicated compound at 0.5 mM (post-incub), and the response reactivated by a second SNP application (2nd). Consistent recovery was seen after vehicle, 5-NMFC, or 5-CMFC but not after incubation with 5-PMFC or VZHE006 indicating ion channel inhibition. The n values are as shown; each line represents a series of recordings from one oocyte.

During the 2 h incubation period (without the cGMP agonist SNP present), the ionic conductance recovered completely, as confirmed in trend plots tracking the conductance values for vehicle saline-incubated individual oocytes through the treatment series (Figure 2). Amplitudes of ion conductances after the incubation period (“post incub”) in all treatment groups were comparable to those in the initial condition, showing responses were uniformly reversible, and ruling out toxicity or cell damage. Second applications of cGMP agonist in normal saline were used to assess the level of block established during the incubation period. The AQP1 conductance was fully reactivated in oocytes incubated in vehicle treatment, showing that repeated recordings were well tolerated. The profound lack of AQP1 reactivation by cGMP after 2 h incubation in 0.5 mM 5-PMFC demonstrated effective block of the ion conductance. VZHE006 caused moderate inhibition, whereas 5-NMFC and 5-CMFC showed no appreciable blocking effect. No current activation by cGMP or effects of furan compounds were seen in non-AQP-expressing controls (see Figure 4 below).

**FIGURE 3:**
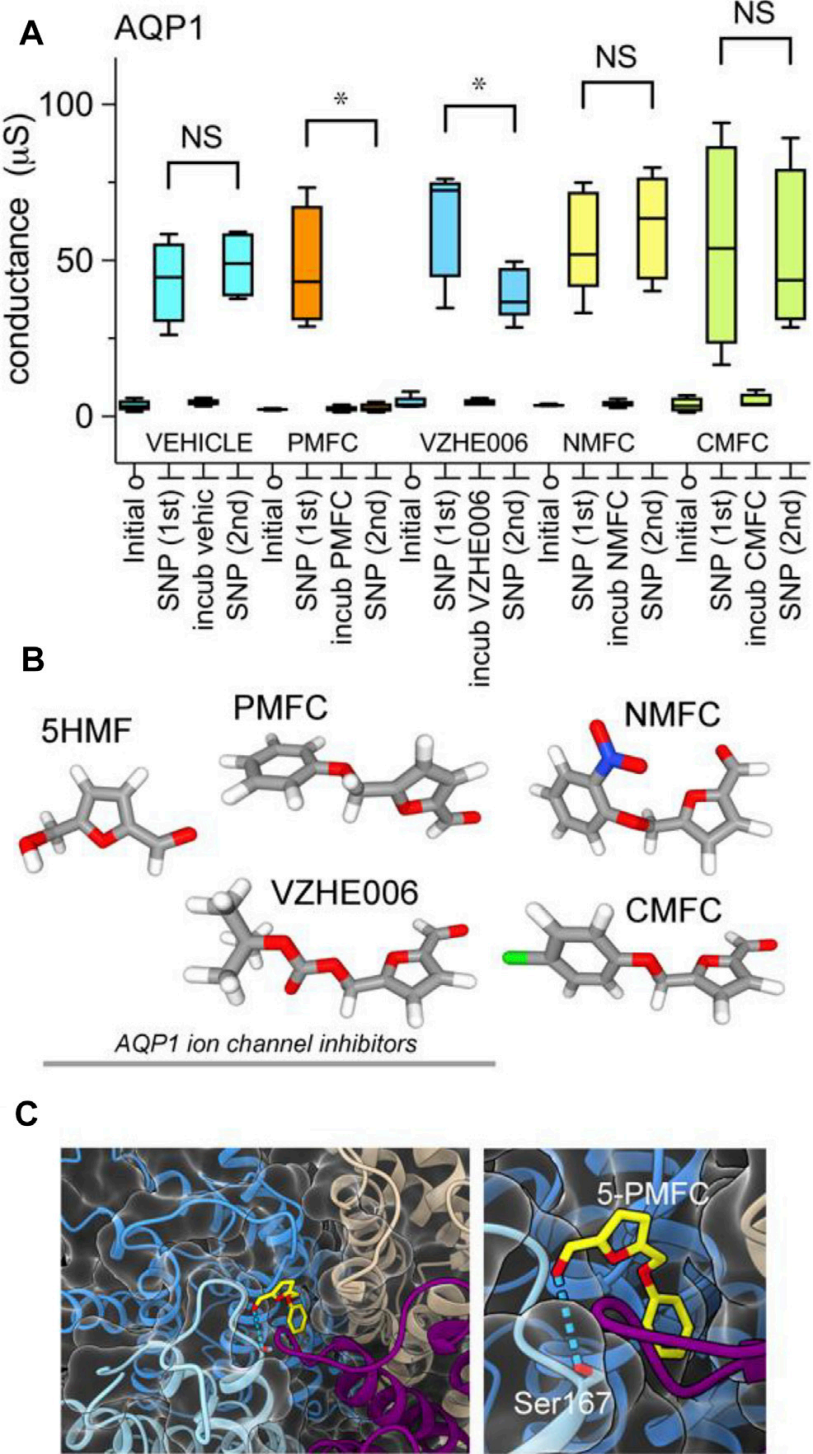
Compiled data showing the effects of furan derivatives on the amplitude of the cGMP-dependent AQP1 ion conductance activated by SNP. **(A)** Compiled box plot data showing statistically significant inhibition of the AQP1 ion conductance by 5-PMFC or VZHE006, but not vehicle, 5-NMFC or 5-CMFC. *n* values were four each for all but 5-NMFC which was 5. Statistical significance was determine by paired Students *t* test; *p < 0.05; NS not significant. **(B)** Space filling structures of the compounds tested; the three on the left act as AQP1 inhibitors. **(C)** In silico docking model illustrating the predicted site for the most favorable interaction of 5-PMFC with AQP1, located near the central pore vestibule of the tetrameric channel in the loop D gating domain, which is seen as a curved purple strand connecting transmembrane helices (left panel). Higher magnification view of the predicted hydrogen bond interaction between 5-PMFC and Ser167 (right panel).

Ion conductance values were calculated from linear slope values from of current-voltage recordings, and compiled in a box plot (Figure 3) and dot plots (Supplementary Figure S1) to evaluate cGMP-activated conductance levels in AQP1-expressing oocytes, and effects of incubation with vehicle or furan compounds. Responses measured from the same oocytes after incubation with 5-PMFC or VZHE006 were significantly reduced as compared to initial responses to SNP used to induce intracellular cGMP signaling (Figure 3A). Structures of the furan derivatives are illustrated in (Figure 3B). Figure 3C shows the results of in silico modeling of the predicted binding site for 5-PMFC on the AQP1 channel, suggesting that the most energetically favourable site for interaction is located at the intracellular side of the central pore of the tetramer, in the gating domain (loop D) at a highly conserved serine residue (serine 167 in human AQP1). The predicted interaction energy of 5-PMFC at this site is −5.1 kcal/mol, which is more favorable than the predicted energy of interaction of 5HMF (−4.9 kcal/mol), consistent with the greater effectiveness of 5-PMFC in blocking the AQP1 ion conductance (Figure 3A). This region in the loop D domain appears to be important for channel activation; mutation of an adjacent highly conserved residue, glycine 166 to proline, was found previously to significantly augment cGMP activation of the AQP1 ion conductance (Kourghi et al., 2018). Additional poses identified by in silico docking for AQP1 and predicted energies of interaction with 5-PMFC are illustrated in Supplementary Figure S2.

**FIGURE 4:**
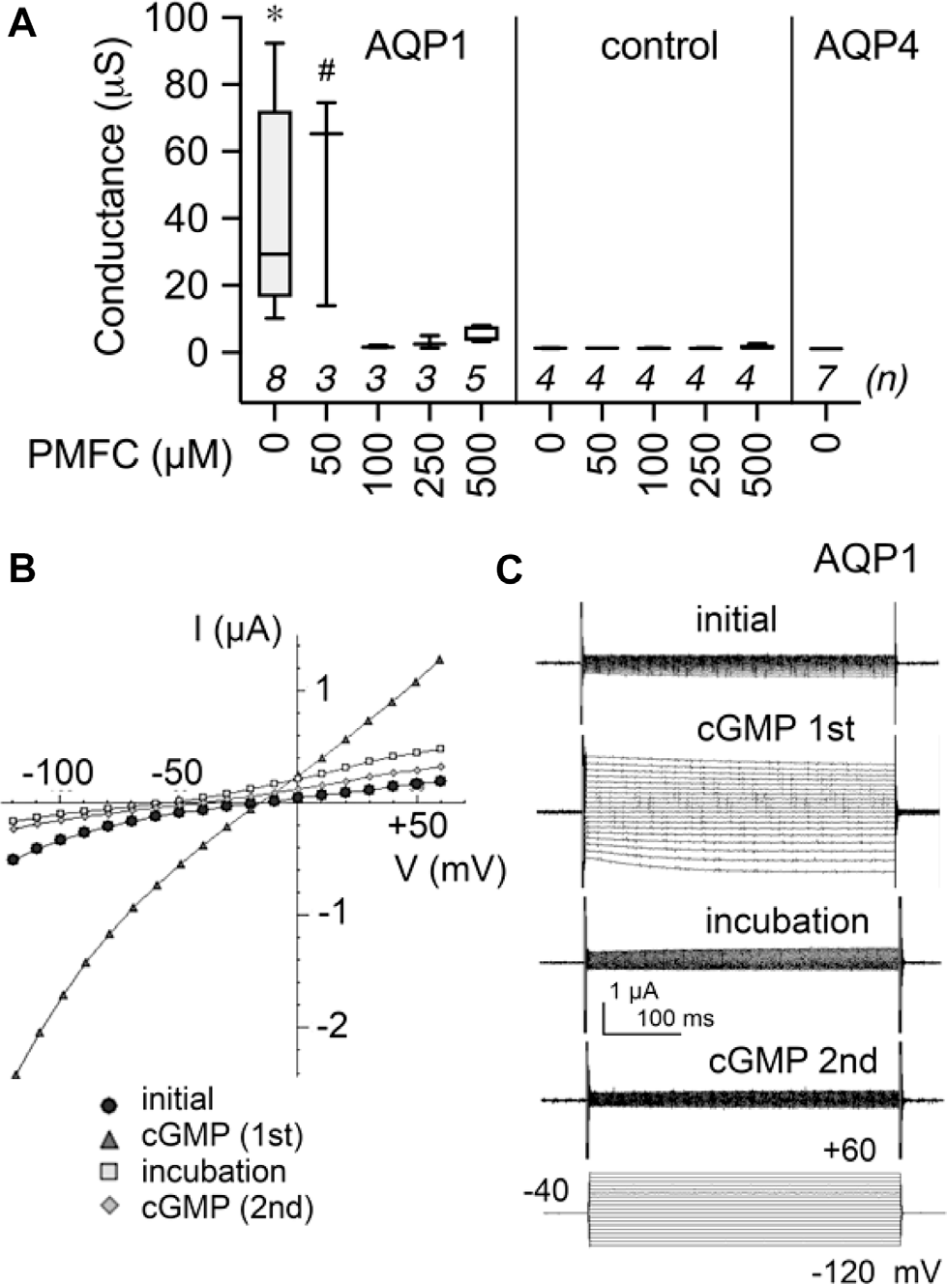
Dose-dependent inhibition of AQP1 ion channel conductance by 5-PMFC. **(A)** Box plot compilation of SNP-activated conductance values measured after 1–2 h incubation with the indicated concentration of 5-PMFC in isotonic saline. Conductances were calculated from slopes of current-voltage (I–V) plots measured at 8 min after application of 3 mM extracellular SNP used to stimulate intracellular cGMP. No current activation by SNP was observed in non-AQP controls with or without 5-PMFC incubation, or in AQP4-expressing oocytes. *n* values in italics are above the *x*-axis. Significant differences were found between AQP1 at 0 µM 5-PMFC (*) and all other treatment groups shown, except AQP1 at 50 µM PMFC (#) which was not significantly different from AQP1 0 µM 5-PMFC. Statistical significance was assessed by one-way ANOVA and Šidák’s multiple comparisons test; *p < 0.01; # not significant. **(B)** Current-voltage plots of responses recorded in series for the same AQP1-expressing oocyte, showing initial low conductance, activation by SNP (‘cGMP 1st′), recovery during 2 h incubation in 500 µM 5-PMFC (incub PMFC), and block of the second response to SNP (‘cGMP 2nd′). Reversal potentials shifted from near −20 mV in the initial and cGMP (1st) conditions to approximately −50 mV after 5-PMFC treatment (in incubation and cGMP (2nd) conditions), consistent with inhibition of a non-selective cation conductance, and not native oocyte K^+^ channels. **(C)** Traces illustrating data shown in the I-V plots in (B).

Figure 4 shows the dose-dependent block of nonselective cation current by 5-PMFC in AQP1-expressing oocytes, with no effect in non-AQP-expressing control oocytes. The boxplot (Figure 4A) shows compiled data for conductance levels activated by 3 mM SNP, measured from slopes of current-voltage plots. AQP1-expressing oocytes showed plateau activation of ion conductances by 8 min after 3 mM SNP application, which was blocked by 1–2 h incubation in isotonic saline with 5-PMFC at doses of 100–500 µM. No significant inhibition was observed at 50 µM. Control oocytes and AQP4-expressing oocytes showed no current activation in response to SNP. AQP4-expressing oocytes had high osmotic water permeability but showed no current activation by SNP, confirming that the capacity for aquaporins to show cGMP-induced ion currents depends on the type of AQP expressed and is not an indirect effect of increased osmotic water permeability in the oocyte expression system. A set of current-voltage plots for a single AQP1-expressing oocyte taken through a series of treatments (Figure 4B) shows low initial current, subsequent activation of the ion current by SNP, recovery to baseline after incubation in saline with 500 µM 5-PMFC, and inhibition of the subsequent current activation by SNP, illustrating channel inhibition. The negative shift in the reversal potential between recordings done before and after 5-PMFC treatment was consistent with block of a non-selective cation channel and not endogenous K^+^ channels. Figure 4C shows traces corresponding to the data shown in the current-voltage plots in Figure 4B.

**FIGURE 5:**
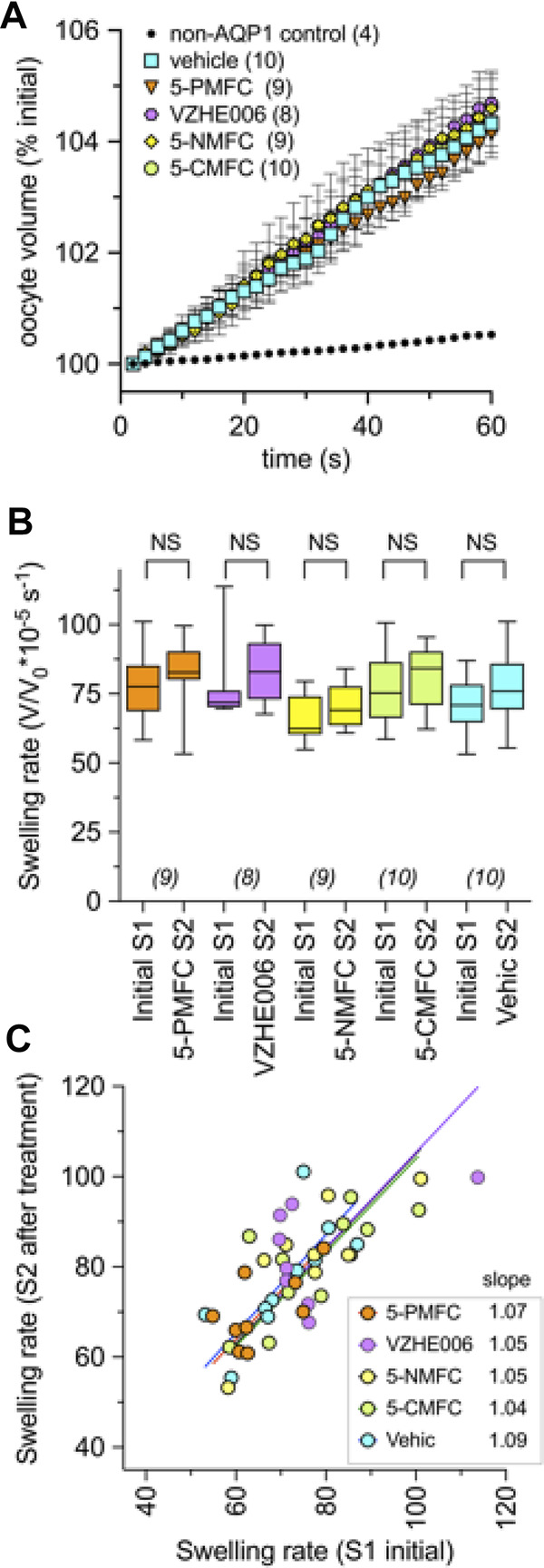
Osmotic water permeability of AQP1-expressing oocytes is not altered by treatment with the furan derivatives. **(A)** Mean swelling responses of AQP1 expressing oocytes in 50% hypotonic saline were not affected after 2 h of preincubation in the furan derivatives (2 mM). Data are mean ± SEM; *n* values are as shown in the key. **(B)** Compiled box plot data showing comparable swelling rates measured in the same oocytes for the first (S1) assay before and second (S2) assay after 2-h incubation in saline with indicated treatments. Statistical significance was assessed by ANOVA and post hoc paired Student *T* tests; NS not significant. **(C)** The plot of S1 vs. S2 swelling rates for individual oocytes shows a linear relationship (slope values near 1.0) in all treatment conditions, indicating no effect on water channel activity, and no change in oocyte membrane integrity or levels of AQP1 channel expression during pharmacological incubation or repeated assays. “Vehic” is vehicle control (equivalent DMSO).

None of the furan derivatives had any effect on AQP1-mediated osmotic water permeability (Figure 5). AQP1 water channel activity depends on intrasubunit water pores rather than the central pore, and has been shown previously to differ from the central pore in pharmacological sensitivities to inhibitors (Yool and Campbell 2012). Non-AQP-expressing control oocytes as expected showed very little osmotic swelling. Oocytes expressing AQP1 after 2 h incubation in saline with the indicated compounds (2 mM) or an equivalent vehicle, were assessed for osmotic water permeability using an optical swelling assay without blocker (Figure 5A). All AQP1-expressing oocytes showed strong swelling in hypotonic extracellular saline. AQP1-mediated swelling showed no effects of vehicle or furan treatments, as summarized in the box plot (Figure 5B). AQP1-expressing oocytes were tested in double swelling assays, in which each oocyte was tested before and after 2 h incubation in isotonic saline containing vehicle or the furan compounds at 2 mM. There were no significant differences between the first (S1) and second (S2) swelling rates for individual oocytes in any of the treatment groups (Figure 5C), as indicated by the slope values near 1.0 in plots of S1 versus S2 swelling rates. These results showed that none of the furan compounds affected AQP1 osmotic water permeability, and also confirmed that the AQP1 channels remained functionally intact and localized in oocyte plasma membrane through the experimental treatments.

**FIGURE 6:**
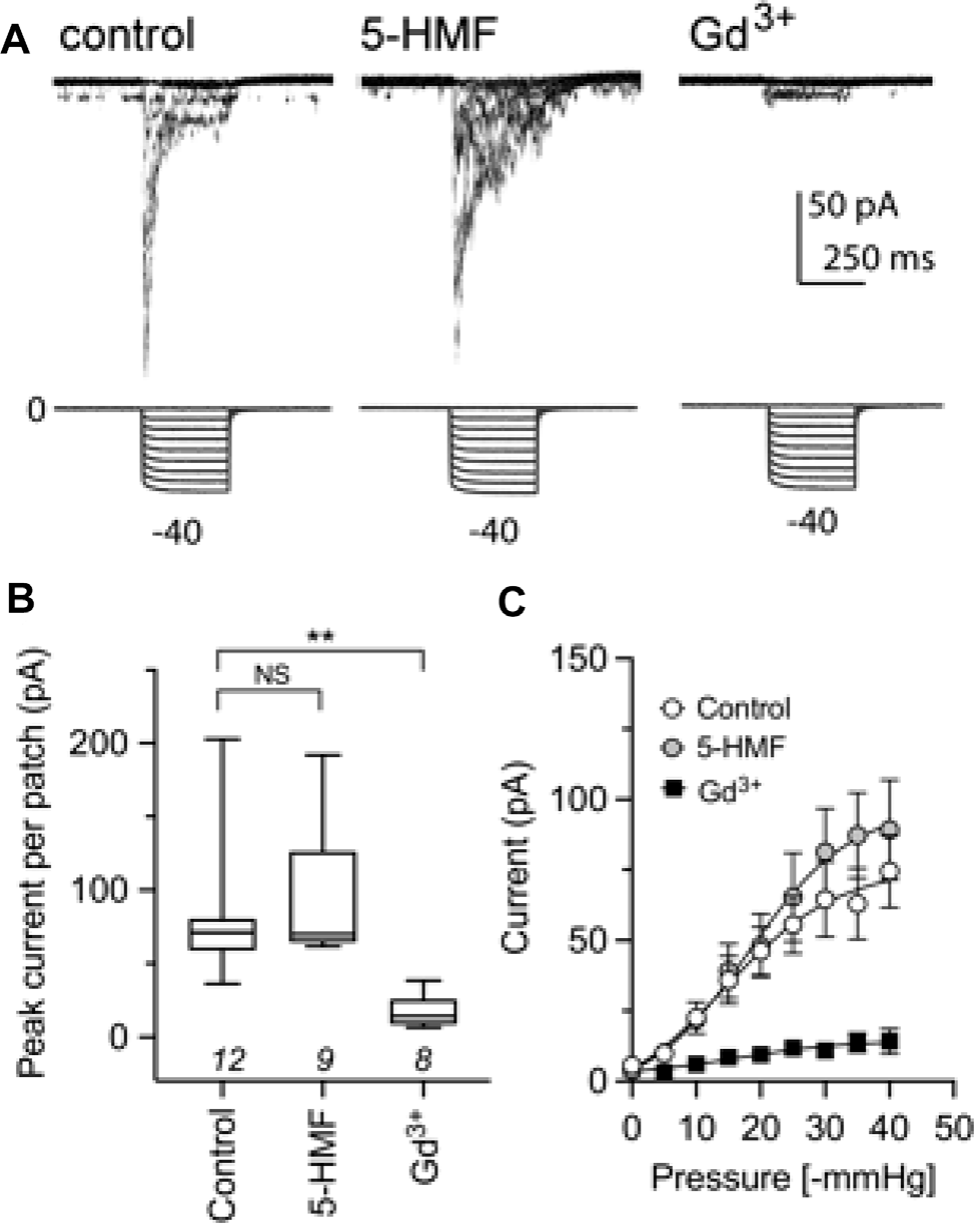
The Piezo1 ion channel conductance is not sensitive to block by 5-HMF. **(A)** Representative traces from cell-attached recordings of human Piezo1 currents (upper row) measured in HEK293T cells stably transfected with Piezo1, shown for cells in the control treatment **(left),** after 2 mM 5-HMF for 60 min (**center),** or after 100 µM GdCl_3_ applied in the bath for ≥5 min before patching **(right).** Square wave pressure pulses (lower row) were applied to the patch pipette using a high-speed pressure clamp. **(B)** Amplitudes of peak currents recorded from cell attached patches from HEK293T cells in control, 5-HMF and Gd^3+^ treatment groups as specified in panel A. ** is *p* < 0.01; NS is not significant; *n* values are above the *x*-axis. **(C)** Pressure-sensitive activation of Piezo1 currents in control, 5-HMF and Gd^3+^ treatment groups. Statistical significance was assessed by Kruskal-Wallis ANOVA and post hoc Dunn’s multiple comparisons tests; **p < 0.01; NS not significant.

Piezo1, a cation channel present in human RBCs and previously proposed to contribute to the Psickle current (Cahalan et al., 2015), showed no effect of treatment with 2 mM 5-HMF in bath saline after 1 h incubation (Figure 6). As expected, Piezo1 current was inhibited within 5 min by the known blocker Gd^3+^ at 100 μM, shown previously to block mechanosensitive ion channels (Yang and Sachs 1989) including Piezo1 (Coste et al., 2010). The Gd^3+^ blocker was added to the bath for Piezo1-expressing HEK cells in advance of cell-attached electrophysiological recording. This protocol achieved full channel block, serving as a positive control. Human Piezo1 channel currents were recorded in cell-attached patches from stably transfected HEK293T cells, and activated by square-wave pressure pulses (Figure 6A). Currents were significantly reduced in amplitude after treatment with Gd^3+^, but not 5-HMF (Figure 6B). 5-HMF did not alter the pressure dependence of Piezo1 for ion channel activation (Figure 6C).

**FIGURE 7:**
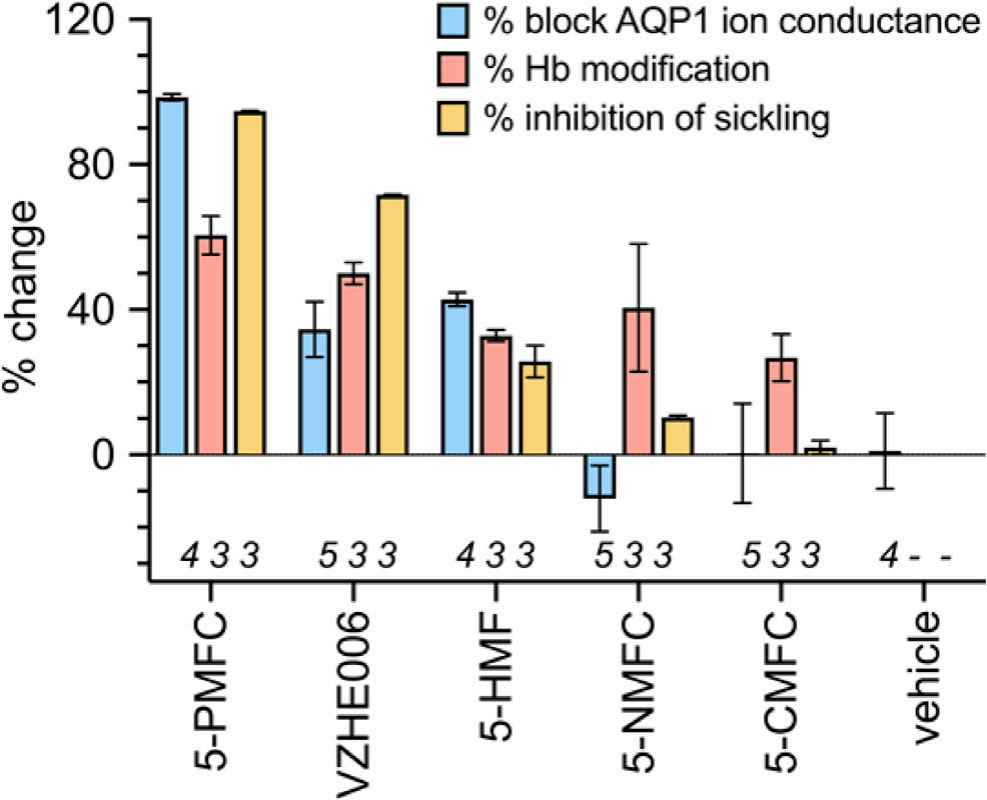
Effects of selected furan compounds on percent block of AQP1 ion conductance amplitudes, percent structural modification of hemoglobin (Hb), and percent inhibition of sickling in SCD red blood cells exposed to low oxygen. Tests of AQP1 block were done with agents at 0.5 mM each; tests for hemoglobin modification and sickling risk were done at 2 mM each. Data for Hb modification and red blood cell sickling were based on results presented in published tables (Xu et al., 2017).

A comparison of the effects of 5HMF and structural derivatives on the amplitude of the AQP1 ion conductance (results here), and the level of hemoglobin modification, and the percentage of SCD cell sickling in hypoxic conditions (summarised from prior work for comparison) is illustrated in Figure 7. The magnitude of sickle cell inhibition at 2 mM was 5-PMFC > VZHE006 > 5-HMF > 5-NMFC > 5-CMFC (Xu et al., 2017). The agents 5-PMFC, VZHE006 and 5-HMF significantly inhibited the AQP1 ion channel at 0.5 mM, whereas 5-NMFC and 5-CMFC were not effective at concentrations up to 2 mM. Interestingly, though 5-PMFC and VZHE006 yielded levels of Hb modification similar to those of 5-HMF and 5-NMFC, these agents did not protect SCD RBCs from sickling, indicating that Hb modification alone does not account for a full beneficial effect. Instead the furan agents showed efficacies consistent with a dependence on both the ability to modify Hb and the ability to block the AQP1 ion channel conductance, supporting a novel dual targeting mechanism.

**FIGURE 8:**
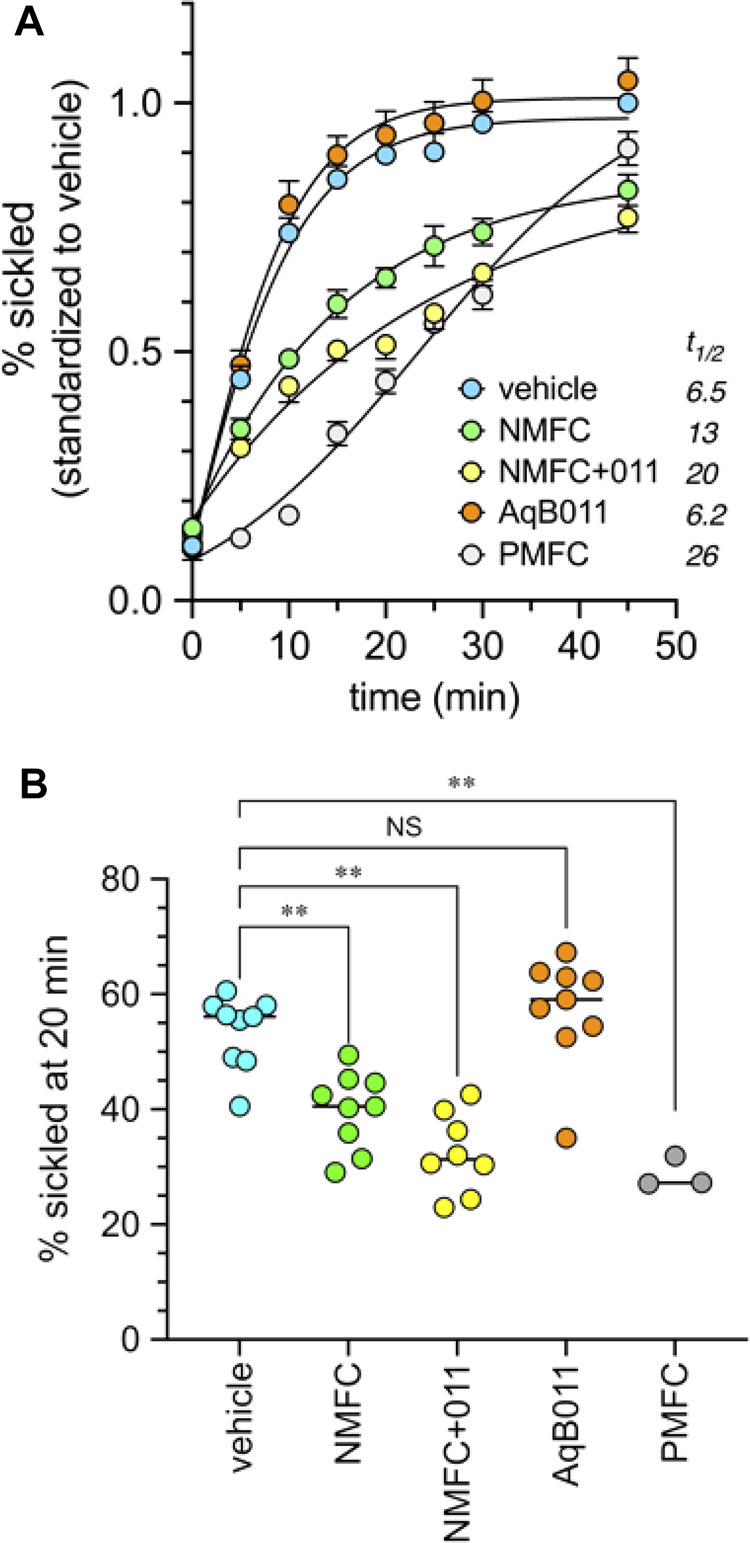
Combined application of agents that inhibit AQP1 ion channels (AqB011) and that modify hemoglobin (5-NMFC) reduce the rate of sickling of human SCD cells in hypoxic conditions, approaching the protection seen with 5-PMFC alone. **(A)** Data for percent sickling were standardized to the mean maximum sickling (at 45 min) measured in the vehicle controls in the same experimental group; data within treatments were averaged for all experiments to determine mean and SEM values, and plotted as a function of time in full nitrogen starting at time zero. Estimated t_1/2_ values (figure legend) show the times needed in minutes to reach 50% of the net maximal sickling level, set as the level observed for vehicle-treated cells at 45 min. Data were compiled from three separate experiments with 2 to 3 replicate samples each. *n* values are as shown in the dot plot below. **(B)** Dot plot summary of raw data for the percentages of sickled cells at 20 min hypoxia compiled from three independent experiments. Concentrations of agents used were 2.5 mM for 5-NMFC and 5-PMFC, 80 µM for AqB011, and equivalent DMSO for the vehicle control. Significant differences were determined by ordinary one-way ANOVA with Šidác’s multiple comparisons test. **p < 0.01; NS is not significant.

The prediction that a combination of AQP1 block and HbS modification should improve protection from sickling over either treatment alone was tested by co-application of AqB011 (a pharmacological inhibitor of the AQP1 ion conductance (Kourghi et al., 2016)), and the furan derivative 5-NMFC which causes Hb modification (Xu et al., 2017) without blocking AQP1. Data in Figure 8 show that the presence of 80 µM AqB011 in combination with 2.5 mM 5-NMFC conferred a significant increase in protection of human SCD red blood cells from sickling during hypoxia. The combination was more effective than either alone. The time (t_1/2_) needed for the proportion of sickled cells to reach 50% of maximum (referenced to the % sickled cells in vehicle treatment at 45 min) differed across the treatment groups (Figure 8A). Rapid onset of sickling was seen in vehicle-treated and AqB011-treated groups (estimated t_1/2_ 6–7 min). The window of protection was doubled with 5-NMFC alone (t_1/2_ 13 min), but was substantially increased in treatment with combined 5-NMFC + AqB011, and treatment with 5-PMFC, for which t_1/2_ values were prolonged 3 to 4 fold over vehicle control (t_1/2_ 20 min or more). The percentages of sickled cells after 20 min hypoxia (Figure 8B) were reduced significantly by coapplication of AqB011 with 5-NMFC, as compared with 5-NMFC alone. 5-PMFC was the most effective of the agents tested in reducing sickling in human SCD cells.

## 4 Discussion

Cellular loss of K^+^, Cl^−^ and water drives dehydration and increases the concentration of HbS in red blood cells of patients with SCD, leading to cell sickling (Joiner 1993; Gibson and Ellory 2002; Lew and Bookchin 2005). Compounds structurally related to 5-HMF were shown previously by Abdulmalik and others to protect SCD cells from sickling, with differences in efficacies thought to be due to levels of modification of HbS and oxygen affinity (Xu et al., 2017). Prior observations that 5-HMF inhibited the Psickle cation leak (Hannemann et al., 2014), and blocked the AQP1 ion conductance (Chow et al., 2020) suggested a novel link with cation conductance pathways. Results here demonstrate that the most effective of the anti-sickling 5-HMF derivatives, 5-PMFC, is the most potent inhibitor of the cationic conductance of AQP1. AQP1 has not previously been considered as a component of Psickle, but results here support it as a strong candidate for the monovalent cation leak component that is pharmacologically distinct from the Piezo1, KCC and Gardos channels, and important as an early step in the dehydration cascade leading to the sickling phenomenon.

Multiple lines of evidence now support the initially controversial concept that AQP1 is an ion channel (Yool et al., 1996; Agre et al., 1997; Yool and Campbell 2012), permeable to Na^+^, K^+^, Cs^+^ and Li^+^ but not divalent cations, as demonstrated by electrophysiology experiments, structure-function analyses, molecular dynamic modeling, and real-time visualization with a photoswitchable optical probe (Anthony et al., 2000; Yool and Weinstein 2002; Yu et al., 2006; Campbell et al., 2012; Kourghi et al., 2016; Kourghi et al., 2017; Pei et al., 2019). Unlike classic cyclic nucleotide-gated channels in sensory cells (Broillet and Firestein 1999), the AQP1 ion channel shows very slow activation and deactivation kinetics in response to cGMP on a time scale of minutes, with slow modal behavior marked by a low probability of initial opening, and long bursts of large conductance openings once channels are activated (Anthony et al., 2000). In heterologous expression systems, only a small percentage of the AQP1 water channels are active as ion channels (Saparov et al., 2001; Yool and Campbell 2012); nonetheless this low proportion has been modeled as being sufficient to impose meaningful changes in net ion transport across membranes (Yool and Weinstein 2002). The proportion of AQP1 available for ion channel gating in the total water channel population is modulated by tyrosine phosphorylation in the carboxyl terminal domain (Campbell et al., 2012) and potentially by other factors such as membrane and cytoskeletal protein interactions (Cowan et al., 2000).

5-HMF is known to decrease sickling of RBCs from disease-affected patients, with effects originally attributed to decreased hemoglobin crosslinking (Safo et al., 2004). Of interest to us was the observation that 5-HMF blocked the Psickle current at doses comparable to those used to reduce RBC sickling (Hannemann et al., 2014). At millimolar concentrations, 5-HMF reduced the Psickle component as measured by Rb^+^ uptake in SCD cells; the magnitude of the monovalent ion flux compared for treated and untreated cells correlated strongly with the percentage of cell sickling. In contrast, Gardos activity showed a small reduction by 5-HMF, and no evidence of direct block (Hannemann et al., 2014). SCD cell membranes show increased permeability to K^+^, Na^+^, Cs^+^, Rb^+^ and Li^+^ (Joiner et al., 1993), and an accumulation of intracellular radiolabelled Ca^2+^ that was attributed to increased membrane Ca^2+^ permeability (Etzion et al., 1993). The concept that Psickle was caused by one class of leak channel was adopted, with the logical caution that a single pathway for both mono- and divalent ions remained unproven (Lew et al., 1997), and thus cation leaks might rely on more than one mechanism (Kaestner et al., 2020). Consistent with the idea of more than one pathway for monovalent and divalent leak currents, deoxygenation-induced fluxes of K^+^ and Na^+^ in sickle cells were found to be blocked by extracellular divalent cations; 2 mM Ca^2+^ reduced Na^+^ influx by approximately 30% as compared to Ca^2+^-free solution, and reduced K^+^ efflux by 10% (Joiner et al., 1995), suggesting Ca^2+^ blocked part of the monovalent cation component directly, with an asymmetry possibly reflecting outward rectification.

RBCs deformed by shear stress showed an increase in Na^+^ influx and K^+^ efflux, not dependent on Ca^2+^ or Cl^−^, without an overall change in cation content (Ney et al., 1990). A typical RBC resting potential around −10 mV (Hoffman and Laris 1984) near the Cl^−^ and HCO_3_^−^ equilibrium potentials would be expected to impose similar driving forces for Na^+^ entry and K^+^ exit through a non-selective cation channel pathway, consistent with the lack of effect of Psickle activity on overall transmembrane osmotic gradients. Dehydration results from progressive intracellular ion loss, which could be driven in part by a Na^+^-leak induced activation of the Na^+^-K^+^-ATPase pump which asymmetrically moves 3 cations out (Na^+^) for 2 cations in (K^+^), in a background of high anion and water permeability (Joiner et al., 1986). The molecular identity of the Psickle current has been elusive. Since human RBCs lack DNA, proteins mediating Psickle could reasonably be assumed to be expressed in all RBCs, where their contribution to normal homeostatic control would be expected to provide some selective advantage, perhaps subtle enough to go undectected in normal conditions, but rising dysfunctionally in SCD cells under hypoxic conditions. A random low-probability channel activation during successive hypoxic events is needed to explain the observed stochastic behavior of Psickle, and the broad distribution of sickle cell phenotypes within RBC populations (Lew and Bookchin 2005).

The mechanosensitive Piezo1 channel was suggested previously as a molecular candidate for the Psickle current, based on observations that Piezo1 is expressed in RBCs, activated by membrane deformation (Cox et al., 2016), permeable to mono- and divalent cations, affected by inherited mutations linked to erythrocyte hydration disorders (Gallagher 2017; Lew and Tiffert 2017), and sensitive to block by a tarantula spider toxin which appeared to partially inhibit Psickle monovalent cation fluxes in deoxygenated conditions, as measured by whole cell patch clamp in SCD cells (Ma et al., 2012). Psickle activation has been associated with Ca^2+^ entry and Mg^2+^ exit (Ortiz et al., 1990; Willcocks et al., 2002). Piezo1 channels are permeable to K^+^, Na^+^, Cs^+^, Ba^2+^, Ca^2+^ and Mg^2+^ (Gnanasambandam et al., 2015). Shear stress increases monovalent cation flux in RBCs (Johnson and Gannon 1990). Piezo1 is gated directly by membrane tension with rapid response times on the order of milliseconds, and is expressed across diverse phyla for feedback control of adaptive responses to environmental physical stressors (Cox et al., 2019). Based on data shown here, the insensitivity of Piezo1 channels to 5-HMF suggests that they do not account for all Psickle properties. We propose the Psickle current is mediated by more than one ion conduction pathway, with Piezo1 positioned as a strong candidate for the divalent cation leak, and AQP1 a logical mechanism accounting for the 5HMF-sensitive monovalent cation leak.

A number of properties of AQP1 ion channels are consistent with a direct contribution to the Psickle conductance. These are: 1) Psickle currents are found in red blood cells, in which AQP1 is expressed (Agre et al., 1995). 2) The Psickle current is blocked by 5-HMF (Hannemann et al., 2014), which also blocks the AQP1 ion conductance (Chow et al., 2020). As shown here, anti-sickling efficacies of 5-HMF-related derivatives correlate with their effectiveness in blocking the AQP1 ion current. 3) SCD cells show elevated levels of cGMP as compared with controls, consistent with an increased activity of the cGMP-dependent AQP1 ion conductance. As measured by enzyme-linked immunosorbent assay (ELISA), cGMP was 6-fold higher in sickle RBCs as compared to control (Conran et al., 2004). In the homozoygous Townes mouse model of SCD, cGMP levels in RBCs were elevated 9- to 13-fold as compared to control animals (Almeida et al., 2020). 4) Psickle monovalent currents are reduced by extracellular Ca^2+^ (Joiner et al., 1995). Multiple classes of AQPs that act as ion channels (including AQP1) show outwardly rectifying block by extracellular divalent cations (1 mM Ca^2+^, Cd^2+^ or Ba^2+^, but not Mg^2+^), suggesting a conserved mechanism across phyla (Kourghi et al., 2017). AQP1 binding sites for divalent cations also have independently been proposed from effects on the AQP1 central pore structure seen in electron cryo-crystallography analyses (Ren et al., 2000) and the presence of a putative candidate Ca^2+^-binding motif in the carboxyl terminal domain (Fotiadis et al., 2002). 5) The stochastic behavior of Psickle suggests a dependence on low-probability channel activation events (Lew and Bookchin 2005). The probability of AQP1 channel opening is low, modulated by tyrosine phosphorylation state, and the kinetics of channel gating are slow (Anthony et al., 2000; Campbell et al., 2012), consistent with a low amplitude background current that might escape detection in normal cells and account for the stochastic nature of Psickle in hypoxic SCD cells.

Considering current drug discovery work for SCD, it is interesting to note that the compound GBT1118 is structurally related to Voxelotor, an approved drug recently shown in the HOPE Phase III clinical trial to be effective in increasing SCD patient hemoglobin concentrations, which in turn correlated with reduced incidence of vaso-occlusive crises (Howard et al., 2021). GBT1118 reduces RBC sickling, increases oxygen affinity, stabilizes HbS structure, and decreases cation fluxes mediated by Psickle, Gardos and KCC pathways (Al Balushi et al., 2019). Psickle (defined as the clotrimazole-insensitive component measured by radiolabelled Rb^+^ entry in low oxygen) was the component most sensitive to block by GBT1118 (with an estimated IC_50_ near 0.6 mM). In the presence of GBT1118, an expected correlation between Psickle activity and Gardos activity was lost (Al Balushi et al., 2019), perhaps suggesting the GBT1118-sensitive Psickle K^+^ current is separate from the Ca^2+^ permeation pathway. The possibility that AQP1 is an unrecognized secondary target of action for Voxelotor is currently being explored.

Results here are the first to identify AQP1 as a molecular candidate for the monovalent Psickle current. In the SCD red blood cell, optimal anti-sickling agents might act in parallel to reduce HbS polymerization (countering morphological deformation), and to block the AQP1 ion channel (countering the Psickle leak). Further optimization of the agents and treatment conditions will be needed, and further analyses of roles for other candidates such as Piezo1 channels are of interest for future work. It is interesting to note the proposal by Eaton and others that beneficial agents do not need to block sickling completely, but should slow the process of sickling to facilitate the successful passage of more RBCs through the microcirculation (Eaton 2020). Our data suggest that combined targeting of AQP1 and HbS might achieve this essential aim, prolonging the window of protection.

In addition to RBCs, AQP1 channels are expressed in endothelial and epithelial membranes of many cells (Ma et al., 1998; Agre 2004; Maeda et al., 2009) including vascular endothelium, which is a key player in SCD vaso-occlusion events. The endothelial cell could be an important additional site of action of AQP1 pharmacological inhibitors. AQP1 merits consideration as a co-target in the development of anti-sickling treatments. Candidate drugs, perhaps similar to 5-PMFC with combined actions on both HbS stabilization and AQP1 ion channel inhibition, could be valuable starting points for generating affordable clinical options for treating sickle cell disease around the world.

## Data Availability

The raw data supporting the conclusion of this article will be made available by the authors, without undue reservation.
