# Supplementary material for: Inhibition of the Aquaporin-1 Cation Conductance by Selected Furan Compounds Reduces Red Blood Cell Sickling
Source: Front Pharmacol. 2022 Jan 17;12:794791. doi: 10.3389/fphar.2021.794791 (PMC8801817; doi:10.3389/fphar.2021.794791)
Supplement: Supplementary file 1 [file Presentation1.pdf]

Supplementary data for *Inhibition of the Aquaporin-1 cation conductance by selected furan compounds reduces red blood cell sickling*

Pak Hin Chow, Charles D Cox, Jinxin V Pei, Nancy Anabaraonye, Saeed Nourmohammadi, Sam W Henderson, Boris Martinac, Osheiza Abdulmalik, Andrea J Yool

Supplemental Figure 1. Dot plots showing initial cGMP-activated conductance values for individual AQP1-expressing oocytes, and the effects of incubation with vehicle or furan compounds on the amplitude of the second cGMP-induced current activation responses. Horizontal lines show median values. (Corresponding box plots for the same data are shown in Figure 3).

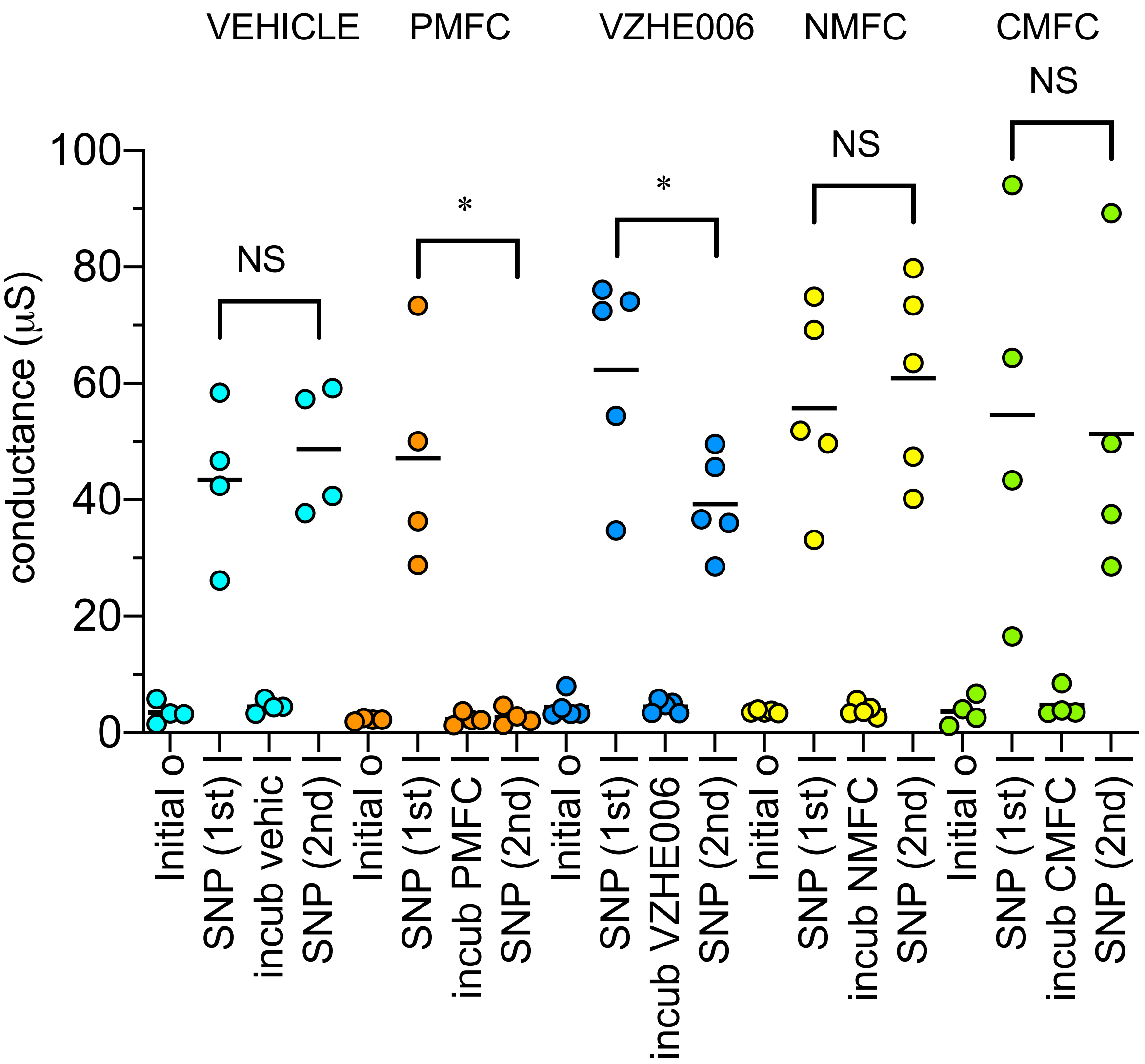

Supplementary data for *Inhibition of the Aquaporin-1 cation conductance by selected furan compounds reduces red blood cell sickling*

Pak Hin Chow, Charles D Cox, Jinxin V Pei, Nancy Anabaraonye, Saeed Nourmohammadi, Sam W Henderson, Boris Martinac, Osheiza Abdulmalik, Andrea J Yool

Supplemental Figure 2. Additional positions detected by in silico modeling as candidate sites of interaction of 5-PMFC across the intracellular face of the AQP1 channel tetramer, with calculated values for the predicted theoretical energies of interaction of the ligand with the channel at the various positions. (See Methods for details.)

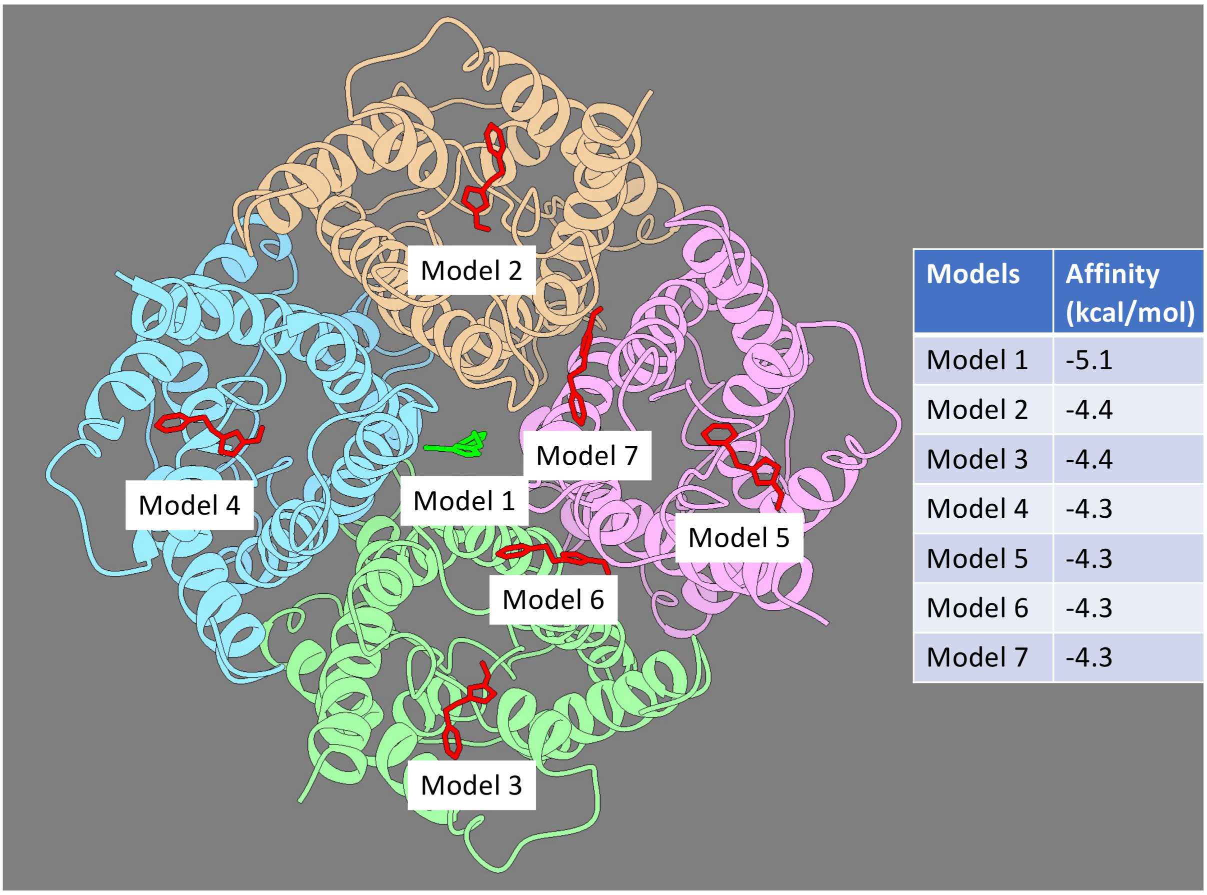

Models	Affinity (kcal/mol)
Model 1	-5.1
Model 2	-4.4
Model 3	-4.4
Model 4	-4.3
Model 5	-4.3
Model 6	-4.3
Model 7	-4.3
